# MAL Daylight Photodynamic Therapy for Actinic Keratosis: Clinical and Imaging Evaluation by 3D Camera

**DOI:** 10.3390/ijms17071108

**Published:** 2016-07-11

**Authors:** Carmen Cantisani, Giovanni Paolino, Giovanni Pellacani, Dario Didona, Marco Scarno, Valentina Faina, Tommaso Gobello, Stefano Calvieri

**Affiliations:** 1 Department of Dermatology, Policlinico Umberto I, “Sapienza” University of Rome, Rome 00185, Italy; paolgio@libero.it (G.P.); dario.didona@gmail.com (D.D.); valentinafaina22@gmail.com (V.F.); stefano.calvieri@uniroma1.it (S.C.); 2 Department of Dermatology, University of Modena and Reggio Emilia, Modena 41121, Italy; giovanni.pellacani@gmail.com; 3 Interuniversity Consortium for Supercomputing (CINECA), Rome 00185, Italy; marco.scarno@gmail.com; 4 Istituto Dermopatico dell’Immacolata (IDI) Hospital, Rome 00195, Italy; to.gobello@alice.it

**Keywords:** actinic keratosis, daylight-mediated PDT, early diagnosis, methylaminolevulinate

## Abstract

Non-melanoma skin cancer is the most common skin cancer with an incidence that varies widely worldwide. Among them, actinic keratosis (AK), considered by some authors as in situ squamous cell carcinoma (SCC), are the most common and reflect an abnormal multistep skin cell development due to the chronic ultraviolet (UV) light exposure. No ideal treatment exists, but the potential risk of their development in a more invasive form requires prompt treatment. As patients usually present with multiple AK on fields of actinic damage, there is a need for effective, safe, simple and short treatments which allow the treatment of large areas. To achieve this, daylight photodynamic therapy (DL-PDT) is an innovative treatment for multiple mild actinic keratosis, well tolerated by patients. Patients allocated to the PDT unit, affected by multiple mild−moderate and severe actinic keratosis on sun-exposed areas treated with DL-PDT, were clinically evaluated at baseline and every three months with an Antera 3D, Miravex^©^ camera. Clinical and 3D images were performed at each clinical check almost every three months. In this retrospective study, 331 patients (56.7% male, 43.3% female) were treated with DL-PDT. We observed a full clearance in more than two-thirds of patients with one or two treatments. Different responses depend on the number of lesions and on their severity; for patients with 1–3 lesions and with grade I or II AK, a full clearance was reached in 85% of cases with a maximum of two treatments. DL-PDT in general improved skin tone and erased sun damage. Evaluating each Antera 3D images, hemoglobin concentration and pigmentation, a skin color and tone improvement in 310 patients was observed. DL-PDT appears as a promising, effective, simple, tolerable and practical treatment for actinic damage associated with AK, and even treatment of large areas can be with little or no pain. The 3D imaging allowed for quantifying in real time the aesthetic benefits of DL-PDT’s increasing compliance.

## 1. Introduction

Photocarcinogenesis is a multistage process that involves initiation, promotion, progression of epidermal dysplastic cells, combined with UV-induced immunosuppression [1,2,3]. Given the increasing incidence of non-melanoma skin cancer (NMSC) worldwide and the recognized risk of actinic keratosis (AK)’s transformation into invasive SCC (even AK grade I, as recently reported) [4], early detection and treatment of AK is needed [1,2,3]. To date, a specific treatment algorithm for first- and second-line therapies is missing, due to the lack of comparative studies. The existing guidelines often lack specific recommendations upon important patient characteristics such as a lesion’s properties and number, the anatomical site involved, the patient’s comorbidities, the devices availability and the dermatologist’s experience [5,6,7,8,9,10,11,12,13]. As it is impossible to predict which AK will transform into invasive SCC, all lesions need to be treated. However, it is important to consider some criteria before deciding which treatment will be indicated for each patient. Several treatments are available including lesion and field-directed options [6,8,10,11,12,13,14,15,16,17]. Among field treatment options, photodynamic therapy has been recognized as a key treatment [6,8,9,11]. Photodynamic therapy is a minimally invasive, highly selective therapeutic modality used for the management of AK and NMSC [6,8]. Conventional treatment using occlusive methyl aminolevulinate (MAL) cream, Metvix^®^; (Galderma Laboratories, Paris, France) for 3 h is an effective procedure for AK and NMSCs. Although conventional (c)-MAL-PDT is a successful treatment for AK, poorer tolerability, long clinic visits, extended treatment times and costly instrumentation limit its wider use [7,12]. To overcome these limitations, Daylight PDT with MAL (DL-PDT) has been developed, showing the same efficacy as c-PDT, with almost no pain and a lower rate of local adverse events [6,7,9,10].

Since 2008, several studies around the world have confirmed efficacy and safety of DL-PDT [5,6,7,8,9,10,11,13]. In our previous study, we retrospectively compared efficacy of both procedures and, due to our city’s geographical location, we were able to perform DL-PDT all year around, giving patients precise instructions to avoid low temperatures (<10 °C) or rainy days during winter. We also helped them spend time under visible light and instructed them to avoid sunburns during summer, which demonstrated similar efficacy [11,18]. The absence of any device such as the lamp (Aktilite^®^ 128, Galderma, Lausanne, Switzerland), and of local skin reactions (LRS), led to high concern about possible relapse. Therefore, it was concluded that showing real-time improvement with 3D imaging may increase compliance.

DL-PDT with MAL has been approved for the treatment of thin, non-hyperkeratotic AK in 2015 in the EU.

A novel device for 3D in vivo optical skin imaging called Antera 3D (Miravex, Ireland), allows the immediate analysis of the optical skin structure to be used. This camera relies on multi-directional illumination and computer-aided reconstruction of the skin surface, illuminating the surface from different angles and using the differences between these images to reconstruct the surface in three dimensions. In this regard, our aim was to evaluate the efficacy of DL-PDT in an objective way.

## 2. Results

From 331 patients evaluated, there was a predominance of male versus female patients (56.7% against 43.3%) (Table 1). The mean age was 73 years old, the average number of lesions per patient at baseline was 4.5 (1–10). Lesion severity was mainly between AK grade KIN (keratinocyte intraepithelial neoplasia) II and KIN III, Fitzpatrick phototype between II and III.

Table 2 shows that the clearance was not dependent on the gender, on the localization of the lesions nor on the phototype. However, a significant relation was found by considering the number of lesions at baseline (*p*-value < 0.001) and for AK severity (*p*-value = 0.003).

Table 3 shows that after the first treatment more than one-third of patients (39.6%) had a full clearance of all AK. More than two-thirds (66.9) reached complete clearance of lesions after two treatments. As already cited, the clearance depends on the number of lesions and AK severity. By considering patients with 1–3 lesions (small cancerization field) at baseline and AK grade I or II, the above percentages became respectively 58.9% and 84.7% after one or two treatments. It has to be noted that all those patients with less than three lesions at baseline and only AK grade I had a full clearance after the first treatment. To better represent such a situation, Table 4 shows that a full clearance was obtained in average after 1.8 treatments (i.e., 3–6 months).

**Figure 1:**
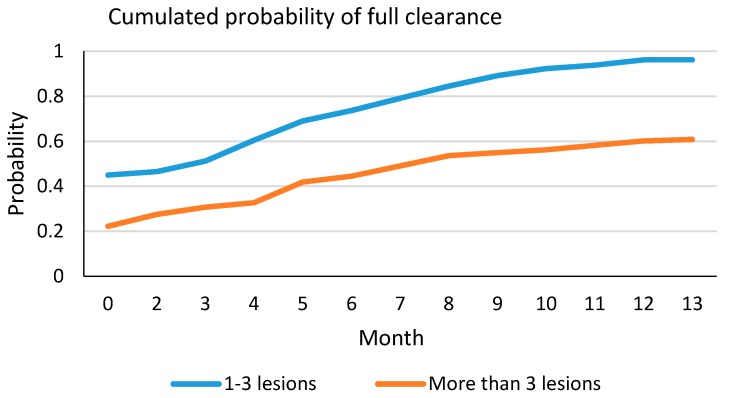
Probability of full clearance for number of lesions, according to the treatment duration (in months).

**Figure 2:**
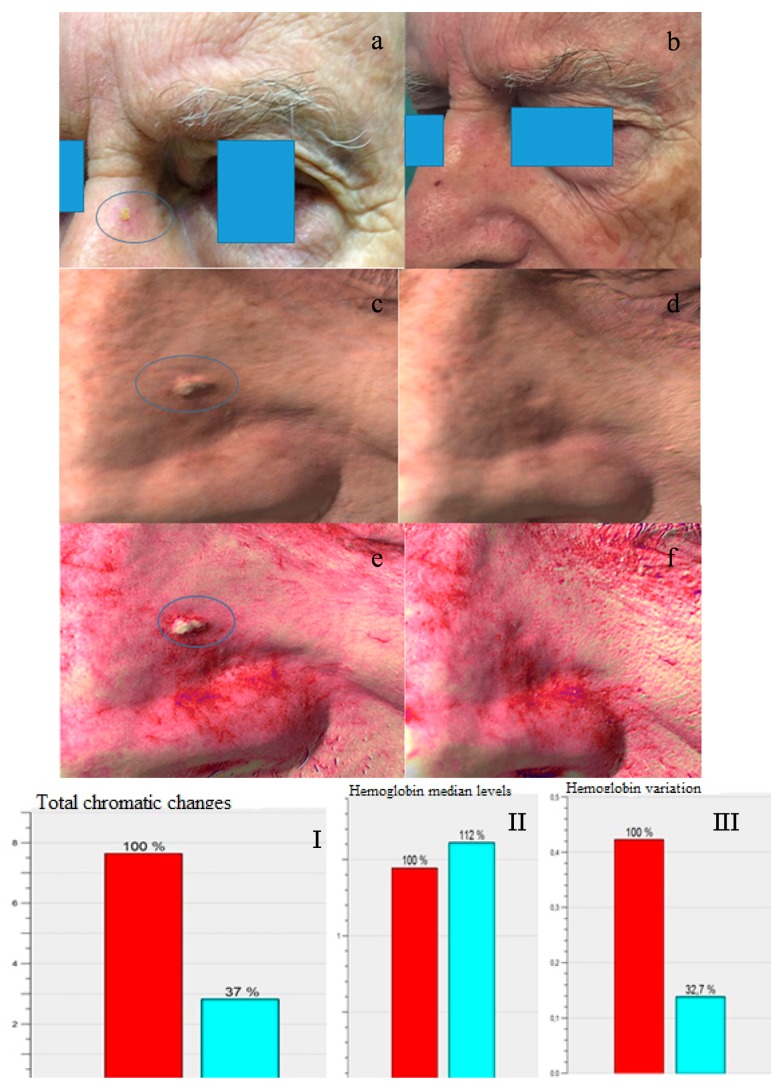
(**a**) Macroscopic picture of KINII AK on the later left side of the nose; (**b**) Complete healing after one MAL-DL-PDT treatment (follow-up after three months); (**c**,**d**) Antera three-dimensional pictures before and after one treatment; (**e**,**f**) Hemoglobin concentration reduction after one treatment; (**I**) The graphs show automated evaluation by Antera software of color and hemoglobin concentration before (red column) and after one treatment (blue column); (**II**,**III**) The graphs show the variation of median levels of hemoglobin before (red column) and after one treatment (blue column). Median level of hemoglobin means median levels of hemoglobin in the area of the lesion; hemoglobin variation means difference in hemoglobin levels before and after therapy.

**Figure 3:**
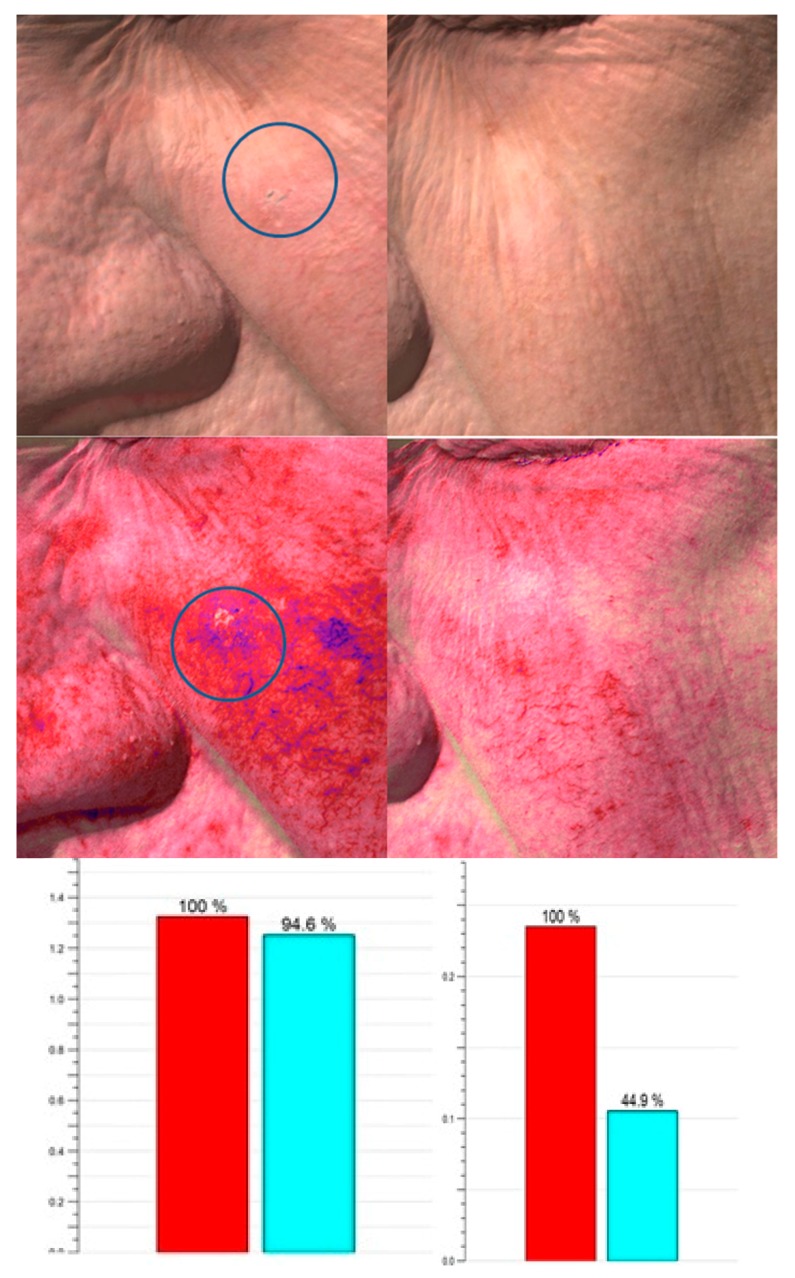
Antera three-dimensional aspect and hemoglobin variation of KINI AK upon examination and after one treatment and automated evaluation. The graphs show the variation of hemoglobin before (red column) and after one treatment (blue column).

**Figure 4:**
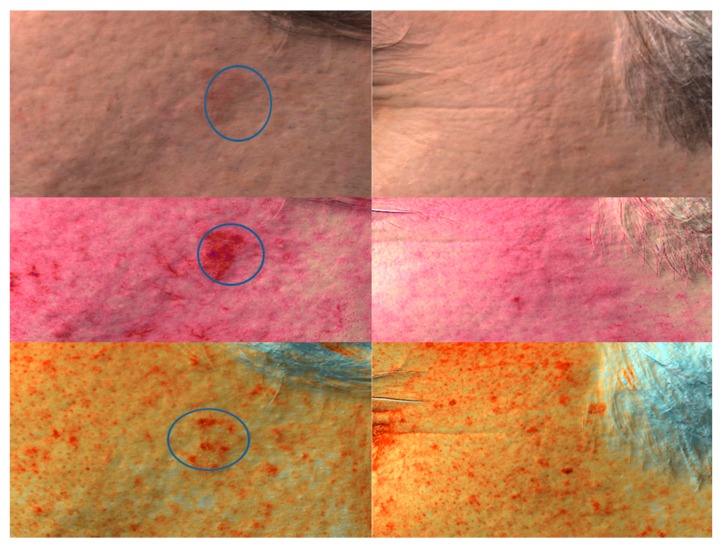
Antera three-dimensional hemoglobin and melanin distribution of AK KIN I before and after one DL-PDT treatment (three months later).

The probability of a full clearance for 1 or 2 (I–II grade) AKs shows a rapid increase in the first two treatments often associated with 3–6 months (Figure 1, Figure 2, Figure 3 and Figure 4; Figure S1), whereas patients with more than four lesions, especially if II–III grade AK, showed a slower response. However, continuing treatments every three months does increase the probability of full clearance.

In our study population, skin reactions were reported by only a small number of patients (5%), especially in those that did not strictly follow our verbal and written information. For example, patients who removed the drug more than three hours after the application likely induced sunburn, especially in the summertime. With regard to local skin reactions, we observed the existence of pain (two points on a median pain scale of 10, burning, itching, cold sensation, erythema and blistering in only three cases (photoallergic reaction). No systemic toxicity was seen or reported. However, all these reactions healed completed without scars, in one or two days using the emollient (such as zinc oxide or hyaluronic acid) that we usually prescribe after treatment and for sun protection. It was also effective for thicker lesions present in the cancerization field, due to keratolytic agents (such as urea 10%–20% or salicylic Vaseline 10%–20%) used two weeks before treatments or a lesion’s preparation by curettage before MAL application.

The 3D imaging device (Antera 3D, Miravex, Limited, Dublin, Ireland) allowed us to objectively assess the clinical response. A general reduction of inflammation (*n* = 310), evaluated with the concentration of hemoglobin seen in the intensity of the red-color intensity has been observed at each visit. (Figure 2 and Figure 3; Figure S1) Furthermore, during the last follow-up, we also observed a general homogenization of the skin color (*n* = 314). Finally, in 309 cases, there was an interesting reduction of wrinkles, observed with a flattening of the cutaneous surface.

Six months later, evaluating each Antera 3D images (Figure 1 and Figure 2) of redness and pigmentation, a skin color improvement or better remodeling in 310 patients was observed. Only in a few cases was a partial increase of redness and melanin found, possibly due to a redistribution of the melanin.

No clinical local recurrence, hyper-pigmented areas nor scars were observed during this study.

Excellent results in terms of complete clearance and excellent cosmetological results were obtained, especially in difficult to treat and poor healing anatomical sites, such as periorbital regions, nose, or ears.

## 3. Discussion

AK is a chronic disease and thus requires an effective, simple, safe solution that allows for the treatment of large areas. DL-PDT is an innovative, simple to perform and almost painless first-line treatment for AKs (able to treat the large fields of actinic damage) [6,7,8,10,13].

In this study, we obtained complete clearance and excellent cosmetological results, especially in difficult to treat anatomical sites and sites of poor healing such as periorbital regions, nose or ears, allowing the patients to recommend it to parents or friends for treatment of AK, as well as aesthetic purposes. In our experience, the combination of DL-PDT with other topical immunomodulators such as imiquimod 5% cream has been shown to be synergistic, increasing effectiveness and helping to achieve a complete clearance of the difficult to treat lesions. Moreover, it may be useful as an adjunctive treatment for some patients to improve outcomes, also in terms of field therapy to treat both the targeted lesions and undetected or subclinical lesions. In this regard, it can be useful as a preventive treatment for NMSCs in transplant patients taking immunosuppressants or with genetic and autoimmune diseases, as well as in immunocompetent patients.

Our study confirms previous reported findings [5,6,7,8,9,10,11], and we were able to automatically quantify the treatment’s effectiveness. Besides the use of automated identification through a vascular pattern, non-invasive devices such as the Antera 3D Miravex camera could be useful in everyday practice, especially for the identification of photo-damaged skin and for therapeutic follow-up. Non-invasive imaging devices in clinical practice and research will help in better diagnosing and treating skin cancers and will be well appreciated by patients. Moreover, these devices offer an objective demonstration of skin changes in real time, especially during therapy with visible light, since the absence of a device such as a lamp, may increase distrust of patients accustomed to attaching the photodynamic treatment to a device and not the ability of a precursor of a photosensitizer (PpIX) selectively inducing cytotoxicity and vascular toxicity activated by a specific wavelength. In our cohort we observed a general reduction of redness (by evaluating the concentration of hemoglobin seen in the intensity of the red color), a general homogenization of the skin color (without hyper-pigmented and/or hypo-pigmented scars), as well as an interesting reduction of wrinkles, observed with a flattening of the cutaneous surface. At this time, we are not able to explain exactly what produces the chromophore changes shown by this device, but they could be due to matrix components of neo-formed collagen re-organization. Nevertheless, automated image analysis could be a useful tool because it eliminates inter-observer variability. Therefore, further studies comparing different devices are needed to standardize diagnosis and care of NMSCs [17].

## 4. Materials and Methods

Patients allocated to our PDT unit (Umberto I hospital, Rome, Italy), affected by single or multiple AKs with grade I and II, subclinical lesions, or fields of actinic damage on sun-exposed areas (scalp, face, neck and hands), were treated with daylight photodynamic therapy (DL-PDT) and were evaluated at baseline and every three months. Although not approved for treatment with PDT (either c-PDT or DL-PDT) when an AK grade III is present in the actinic field, they were treated albeit to lower response rates and with the need for repeated or adjuvant treatments. Patients were verbally informed about the treatment procedures, the benefit limits, the side effects, the alternative treatment options and then all of them signed the consent form before the procedure, allowing to take first macroscopic and then Antera 3D, Miravex^©^ camera pictures of the lesions. Exclusion criteria were: patients with porphyria, or allergic to porphyrins or the other substances in the photosensitizer, to soy, to peanuts, pregnant women and patients using other photosensitizers. The study was performed as an analysis produced by an extrapolation of data coming from our routine medical records.

For all treated patients, skin lesions were recorded before scales were scraped off with a curette. The 16% MAL cream (Metvix^®^, Galderma, Paris, France), which is the only product approved in Italy for PDT treatment of NMSCs and the only one approved with DL-PDT for AK, was directly applied without sun blockers in an approximately 0.5–1 mm thick layer over and around the lesion area (cancerization field). Patients were advised to remain indoors within 30 min after MAL application, before going outdoors under daylight continuously for at least 90 min (if it was a sunny day) or for 120 min under daylight (if overcast or partially sunny day). After visible light exposure, the patient was instructed to wash off the MAL cream with thermal spring water, and apply a pain-relief cream to reduce the itching and burning sensations. On the treatment day, the patients exposed themselves to the daylight between 10:00 and 12:00 in a location about 21 meters above sea level and 41°54′39” latitude North (Rome, Italy) in winter, though earlier in summer to avoid sweating. The final outcome was evaluated clinically, taking into account also the patient’s perception and macroscopic pictures.

Moreover, we used a novel device allowing the immediate analysis of the optical skin image (Antera 3D, Miravex, Limited, Dublin, Ireland). The acquired spectral data was used to map the distribution and concentration of melanin and hemoglobin. Unlike traditional imaging techniques, where only three color channels (red, green, and blue) are used, the Antera 3D^®^ uses reflectance mapping of seven different light wavelengths spanning the entire visible spectrum. This allows for a much more precise analysis of the skin colorimetric properties, which are mostly determined by two dominant chromophores: melanin and hemoglobin. Acquired spectral images are transformed into skin spectral reflectance maps, and the skin surface shape is used to compensate for light intensity variation due to the varying direction of incident illumination. The reflectance data is transformed into skin absorption coefficients and used to quantify melanin and hemoglobin concentrations using mathematical correlation with known spectral absorption data of these chromophores. The hemoglobin levels gave us information about the level of inflammation (directly proportional to the vascularization degree), which corresponded to a difference in red color intensity. The evaluation of the wrinkles was analyzed according to the presence and intensity of cutaneous folds and the relative flattening of the skin. The pictures (before and after the treatment) were taken with the above-cited software (Antera 3D, Miravex, Limited, Dublin, Ireland) facilitating the analysis with an error less or equal to ±5%.

All baseline characteristics were recorded in our medical data base, including patients’ gender and age, as well as the number of initial lesions, the localization (also concerning nose or hairs), the lesion severity (AK grade I–III) and Fitzpatrick photo-type (that, in our cases, was limited to the range 1–3) [14]. Note that these last two variables were considered also as numbers, in order to evaluate their synthesis. This data base allowed us to verify the final clearance (that assumed the values of “partial” or “full”), and the number of treatments.

A total of 331 patients were enrolled in our study; in a first step we recorded information such as their ages, number of baseline lesions, AK severity, and photo-type. We verified that these variables were equally distributed among the patients by considering their gender. To this purpose we used the Student’s *t* test or ANOVA.

Then, assuming that the effects of the variables were constant over time, we verified the number of treatments needed to reach a full clearance, and which factors were associated with it. In this last case, we referred to a chi-square test (note that, in this case, AK severity and phototype Fitzpatrick I–III were considered as ordinal variables).

Finally, we evaluated the relation between the effectiveness of the treatment by considering the mean number of treatments and the months since baseline. We also repeated this analysis by means of a graphical representation, in which we plotted the clearance probability among the months (depending on the initial number of lesions).

In all the cases we considered significant a *p*-value under 0.05.

## 5. Conclusions

Multiple treatments may be needed in a patient’s life as actinic keratosis (AK) is a chronic and relapsing disease. According to modified grading for AK, iSCC can originate from AK with atypia limited to the basal layer [4], and direct origin from a cancer field cannot be excluded. Therefore an effective and prompt treatment of larger areas is needed. Although C-PDT is a successful treatment for AK, poorer tolerability, a longer clinic visit, extended treatment times and costly instrumentation limit its wider use. As a solution for overcoming these limitations, DL-PDT has begun to spread worldwide. Our results confirm data coming from the literature, considering DL-PDT a promising, effective, safe and convenient alternative well accepted by patients for the treatment of AKs. It is suitable for patients with AK grade I and II, subclinical lesions, or fields of actinic damage on sun-exposed areas as a first-line treatment [18]. Although PDT is not approved for AK grade III, in our opinion, they can be partially treated with lower response rates and the need for repeat or adjuvant treatments as shown in by Table 4 [5,6,7,8,9,10]. Close monitoring of elderly or immunocompromised patients with severe fields of actinic damage is highly recommended for early detection and treatment of non-melanoma skin cancer (NMSC). This may allow for reductions in annual treatment cost, especially in terms of access to the surgery room [1,3]. Improved cosmetic appearance and greater patient satisfaction is giving to DL-PDT an aesthetic significance which is increasing patient demand for treatment [8,10,13]. In conclusion, Antera 3D is a valid device to better identify AKs and evaluate the efficacy of the treatment with DL-PDT, showing chromatic (e.g., redness and brown) and structural (e.g., wrinkles) changes such as hemoglobin concentration reduction, remodeling of melanin distribution, probably related to matrix components of neo-formed collagen re-organization, reducing wrinkles and improving skin tone, which can be useful as a general assessment of the therapeutic response, as well as for expanding the field of application of DL-PDT. Further studies are needed to extend the knowledge and the possible applications of this therapeutic treatment and of new devices.

## Figures and Tables

**Table 1:** Mean age, average number of lesions, keratinocyte intraepithelial neoplasia (KIN) and photo-type of all patients and for their gender.

Variables	Patients	Age (Mean and STD)	Lesions (Mean and STD)	KIN (Mean and STD)	Photo-Type (Mean and STD)
Male	188 (56.7%)	73.5 (11.1)	4.6 (2.7)	2.3 (0.6)	2.5 (0.53)
Female	143 (43.3%)	72.3 (8.6)	4.4 (1.8)	2.4 (0.6)	2.4 (0.52)
*p*-Value associated to the TTEst statistic		0.55	0.38	0.3	0.22
Total	331 (100%)	73 (10)	4.5 (2.4)	2.3 (0.6)	2.5 (0.5)

STD means standard deviation; TTEst means statistical *t*-test.

**Table 2:** Value of the χ-square index and of its associated *p*-value for full or partial clearance and gender, class of lesions, localization of lesions, KIN and photo-type.

Test on Dependency between Clearance and:	Gender	Class of Lesions (1–3; 4–6; More than 6)	KIN	Photo-Type
χ Square	0.02	55.4	11.84	1.04
*p*-Value	0.8	<0.001	0.003	0.59

**Table 3:** Follow-ups and percentages of patients for clearance.

Response to Treatment	Number of Follow-Ups			
	1	2	3	4 or More
Partial clearance	8.8	6.6	5.4	5.4
Full clearance	39.6	27.3	5.7	1.2
Full clearance (detail for 1–3 lesions and KIN I–II)	58.9	25.8	11.4	3.9

**Table 4:** Mean number of follow-ups and duration of the treatments (with the corresponding standard deviation) for clearance, with the associated value of the *t*-test statistics.

Clearance	Number of Follow-Ups (Mean and STD)	Number of Months (Mean and STD)
Partial	2.6 (1.6)	5.3 (4.3)
Full	1.8 (1)	3.1 (3.2)
Value of *t*-test statistic and its associated *p*-value	4.4 (<0.001)	3.2 (<0.001)

## Supplementary Materials

Supplementary materials can be found at http://www.mdpi.com/1422-0067/17/7/1108/s1.
