# Supplementary material for: MAL Daylight Photodynamic Therapy for Actinic Keratosis: Clinical and Imaging Evaluation by 3D Camera
Source: Int J Mol Sci. 2016 Jul 11;17(7):1108. doi: 10.3390/ijms17071108 (PMC4964483; doi:10.3390/ijms17071108)
Supplement: Supplementary file 1 [file ijms-17-01108-s001.pdf]

Supplementary Materials: MAL Daylight Photodynamic Therapy for Actinic Keratosis: Clinical and Imaging Evaluation by 3D Camera

Carmen Cantisani, Giovanni Paolino, Giovanni Pellacani, Dario Didona, Marco Scarno, Valentina Faina, Tommaso Gobello and Stefano Calvieri

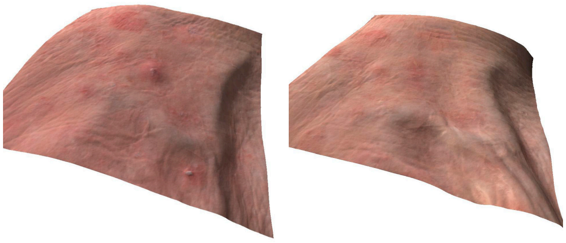

(**A**)

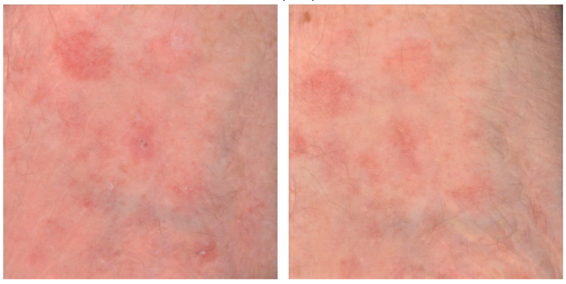

(**B**)

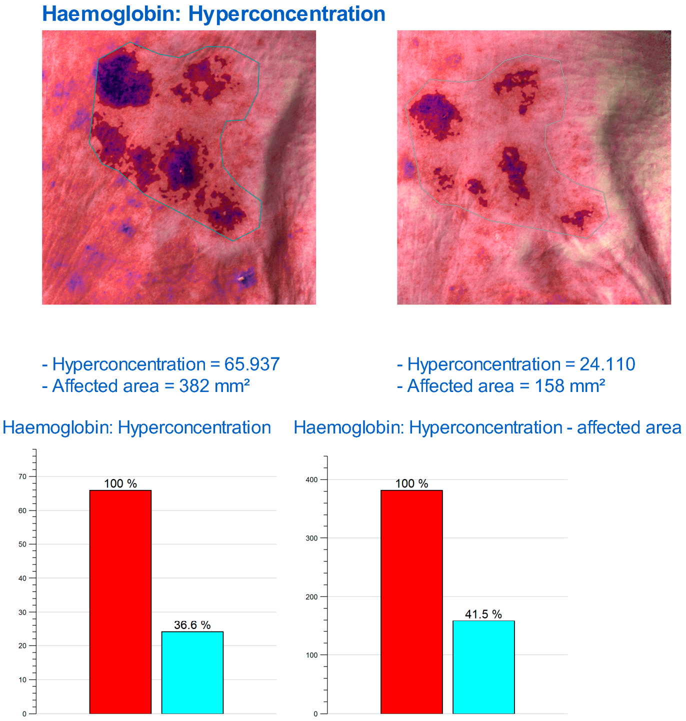

(**C**)

Figure S1. *Cont.*

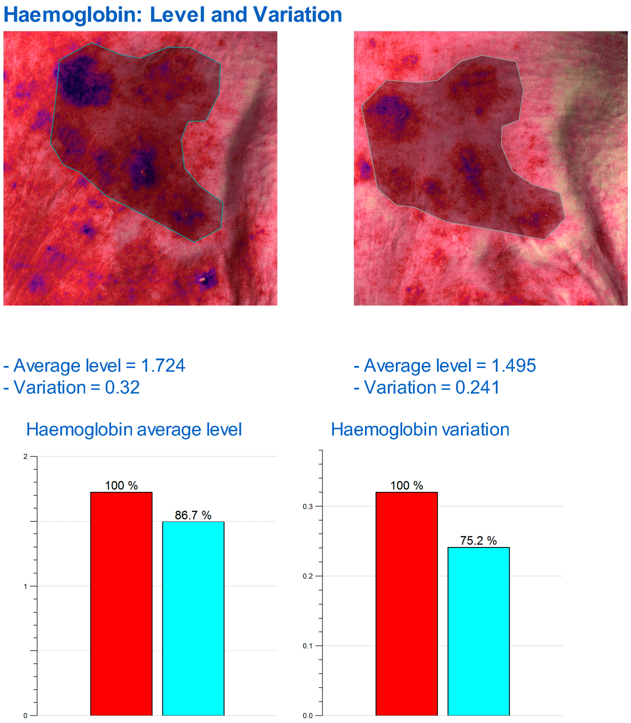

(**D**)

Figure S1. (**A**) Multiple actinic keratoses highlighted by Antera 3D, Miravex; (**B**) A particular of the same actinic keratoses; (**C**) Hemoglobin concentration in the affected area and variation of hemoglobin before and after one treatment. The **left** figures are before the treatment, while the **right** figures are after the treatment. In the graphs there is represented the variation of hemoglobin before (red column) and after one treatment (blue column); (**D**) Hemoglobin concentration in the affected area and variation of hemoglobin before and after one treatment. The **left** figures are before the treatment, while the **right** figures are after the treatment. In the graphs there is represented the variation of hemoglobin before (red column) and after one treatment (blue column).
